# Supplementary material for: Improving Care Transitions for Hospitalized Veterans Discharged to Skilled Nursing Facilities: A Focus on Polypharmacy and Geriatric Syndromes
Source: Geriatrics (Basel). 2019 Feb 9;4(1):19. doi: 10.3390/geriatrics4010019 (PMC6473365; doi:10.3390/geriatrics4010019)
Supplement: Supplementary file 1 [file geriatrics-04-00019-s001.pdf]

Article

Improving Care Transitions for Hospitalized Veterans Discharged to Skilled Nursing Facilities: A Focus on Polypharmacy and Geriatric Syndromes

Amanda S. Mixon [1,2,3,4,*], Vivian M. Yeh [3], Sandra Simmons [2,3,4,5], James Powers [2,4,5], Eugene Wesley Ely [2,4], John Schnelle [2,3,4,5] and Eduard E. Vasilevskis [1,2,3,4]

1 Section of Hospital Medicine, Vanderbilt University Medical Center, Nashville, TN 37203, USA; ed.vasilevskis@vumc.org
2 Geriatric Research Education and Clinical Center (GRECC), VA Tennessee Valley Healthcare System, Nashville, TN 37212, USA; sandra.simmons@vumc.org (S.S.); james.powers@vumc.org (J.P.); wes.ely@vumc.org (E.W.E.); john.schnelle@Vanderbilt.Edu (J.S.)
3 Center for Clinical Quality and Implementation Research, Vanderbilt University Medical Center, Nashville, TN 37203, USA; vivian.m.yeh@vumc.org
4 Center for Quality Aging, Vanderbilt University Medical Center, Nashville, TN 37203, USA
5 Division of Geriatrics, Vanderbilt University Medical Center, Nashville, TN 37232, USA
* Correspondence: Amanda.S.Mixon@vumc.org; Tel.: +1-615-936-3710

Received: 30 December 2018; Accepted: 5 February 2019; Published: 9 February 2019

Supplementary Materials:

1. Selected assessments

Incontinence assessment

Date of assessment	MM/DD/YYYY
Bladder	0, Continent
	1, Incontinent
	2, Not applicable

If ‘not applicable’ was selected, please elaborate. [free text]

Source of bladder information?	1, Chart
	2, Nurse
	3, Other

If ‘other’, please indicate source. [free text]

Bowel	0, Continent
	1, Incontinent
	2, Not applicable

If ‘not applicable’ was selected, please elaborate. [free text]

Source of bowel information? 1, Chart

2, Nurse

3, Other

If ‘other’, please indicate source. [free text]

Questions to ask patient and/or family:

a. Do you go to the bathroom by yourself? Y/N

b. Do you feel safe going by yourself? Y/N

c. Do you have any urine or bowel accidents? Y/N

If 'yes,' which? 1, Bladder

2, Bowel

3, Both

If 'yes,' did that start in hospital or shortly before you came to hospital (as opposed to long-term)? Y/N

d. Do you want to be assisted to the toilet by a nurse every 2-3 hours? Y/N

Questions to ask the nurse:

Does the patient spontaneously request pain medication on his/her own, if needed?

1, No

2, Yes

3, Sometimes

4, Not sure

5, Pt is non-verbal

6, Pt has no pain (N/A)

a. Does patient try to use the bathroom without asking for assistance? Y/N

b. Can they get to the bathroom safely without assistance? Y/N

c. If they need assistance, how much assistance do they need (e.g., supervision only, limited physical assistance, extensive physical assistance, etc.)? [free text]

Nurse available to answer incontinence questions? Y/N

Please select the source for the information contained above (check all that apply):

1, Patient

2, Family

3, Medical record

4, Nurse/other provider

Nutrition assessment

Date of assessment MM/DD/YYYY

Is there at least one weight recorded in the chart? Y/N

Weight 1: Most recent chart weight (in pounds)

Weight 1 date MM/DD/YYYY

(other weights up to 5 are included)

Any comments regarding weights [free text]

Source of information:
1, Patient

2, Other (family, caregiver, etc.)

3, Both patient and family/caregiver

What does patient/family report for current weight? [free text]

Weight loss prior to hospitalization: Has the patient lost weight without trying to within the last month prior to being admitted to the hospital?
1, Yes

2, No

3, Do not know

If 'yes,' how much? If he/she does not know the amount, type "DK" in the box. [free text]

Weight loss during hospitalization: Has the patient lost weight during this hospital stay?

1, Yes

2, No

3, Do not know

If 'yes,' how much? If he/she does not know the amount, type "DK" in the box. [free text]

Appetite: Have you noticed any change in your/your relative's appetite since being admitted to the hospital?
1, Yes, Decrease in appetite - eating less than normal

2, No change in appetite - eating about the same as normal

3, Yes, Increase in appetite - eating more

Does patient/family think they need either verbal prompting or physical assistance to eat?

1, Verbal prompting/encouragement

2, Physical assistance

3, No assistance required

4, N/A (feeding tube)

Do you have any concerns about your/your relative's eating habits or weight? Y/N

If 'yes,' what are your concerns? [free text]

Is there anything else you would like to share with me about your/your relative's eating habits or weight? [free text]

Would this patient be a good candidate for observation of meal intake? Y/N

If 'yes,' why (e.g., low weight, discrepancy between chart documentation of weight and/or intake and patient/family report, etc.) [free text]

Figure S1 Nursing Transition Summary (NuTs)

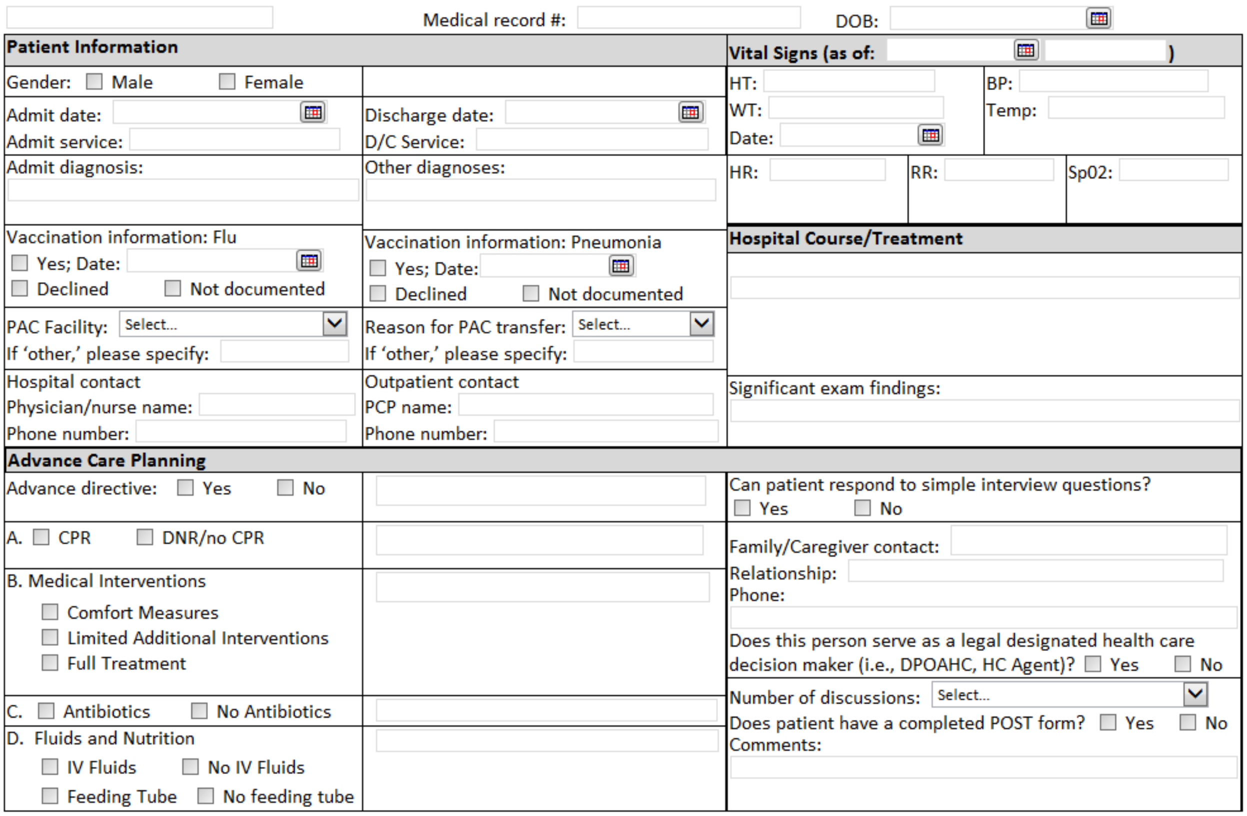

Medical record #: DOB:

Patient Information

Gender: ☐ Male ☐ Female

Admit date:
Admit service:

Discharge date:
D/C Service:

Admit diagnosis:

Other diagnoses:

Vaccination information: Flu
☐ Yes; Date:
☐ Declined ☐ Not documented

Vaccination information: Pneumonia
☐ Yes; Date:
☐ Declined ☐ Not documented

PAC Facility: Select...
If 'other,' please specify:

Reason for PAC transfer: Select...
If 'other,' please specify:

Hospital contact
Physician/nurse name:
Phone number:

Outpatient contact
PCP name:
Phone number:

Vital Signs (as of:)

HT:
WT:
Date:
BP:
Temp:
HR:
RR:
Sp02:

Hospital Course/Treatment

Significant exam findings:

Advance Care Planning

Advance directive: ☐ Yes ☐ No

A. ☐ CPR ☐ DNR/no CPR

B. Medical Interventions
☐ Comfort Measures
☐ Limited Additional Interventions
☐ Full Treatment

C. ☐ Antibiotics ☐ No Antibiotics

D. Fluids and Nutrition
☐ IV Fluids ☐ No IV Fluids
☐ Feeding Tube ☐ No feeding tube

Can patient respond to simple interview questions?
☐ Yes ☐ No

Family/Caregiver contact:
Relationship:
Phone:

Does this person serve as a legal designated health care decision maker (i.e., DPOAHC, HC Agent)? ☐ Yes ☐ No

Number of discussions: Select...
Does patient have a completed POST form? ☐ Yes ☐ No
Comments:

Cognitive and Mental Status		Issues to consider
Dementia	Does this patient have a dementia diagnosis? ☐ Yes ☐ No	
Delirium	Delirium at any point during hospital stay: ☐ Yes ☐ No<br>Onset/Duration:<br>Treatment/Response to treatment:<br>Delirium on discharge: ☐ Yes ☐ No	
Cognitive Status	Was there a change in cognitive status during acute care stay? ☐ Yes ☐ No<br>BIMS score:<br>If 'yes,' please describe:	
Mental Status	Mental Status: Select...<br>Mental Health: ☐ Depression ☐ Anxiety<br>Depression Assessment (e.g., symptoms endorsed, etc.):<br>Treatment/Response to treatment:	
**Functional Status**		**Issues to consider**
Ambulation	Ambulation at discharge: Select...<br>Devices:<br>Does this constitute a change in ambulation from admission or prior to admission? ☐ Yes ☐ No<br>If 'yes,' please describe change in ambulation:	
Transfers	Transfer (including toileting): Select...<br>Devices:	
Weight bearing status	Left: Select...<br>Right: Select...<br>Additional information regarding weight bearing status:	
Fall risk	Is patient a fall risk? ☐ Yes ☐ No<br>Additional information (including restraints used, recent falls, etc.):	
Bowel	☐ Continent<br>☐ Constipated ☐ Incontinent<br>Last BM:<br>If 'incontinent' or 'constipated,' additional information:	
Bladder	☐ Continent<br>☐ Incontinent ☐ N/A (catheter)<br>Voiding frequency during last day in acute care:<br>Source: Select...<br>If incontinent, please select: Select...<br>Additional information/instructions:	
Catheter	Did patient have a Foley Catheter during hospitalization: ☐ Yes ☐ No<br>Date placed:<br>Reason:<br>Complications:<br>Date removed: ☐ N/A<br>Did patient void after f/c removed: ☐ Yes ☐ No<br>Instructions: ☐ Do not remove<br>☐ Change monthly<br>☐ Other:	
**Nutrition**		**Issues to consider**
Tube feeding	Was patient discharged with a feeding tube? ☐ Yes ☐ No<br>If 'yes,' type:<br>If tube feeding, insertion date:	Instructions/supplements:
Oral food/fluid intake	Speech/swallow eval? ☐ Yes ☐ No If 'yes,' significant findings:<br>Feeding assistance required: Select...<br>Estimate of intake on last day in acute care: Select...<br>Instructions/supplements if intake is below 50%:	Diet:

**Nursing**		**Issues to consider**
Pain	Range of pain during course of hospitalization:<br>Pain level on discharge (between 0-10): Select...<br>Worst pain in past 5 days: Select...<br>Location(s) of pain:<br>Treatment/response to treatment (including # of PRN dosages in last 24 hrs):<br>Additional comments regarding pain (Trend, PT/OT notes, etc.):	
Allergies	☐ NKDA	List:
IV access	IV access: Select...	
Devices	☐ Colostomy<br>☐ Urostomy<br>☐ Pacemaker/ICD<br>Additional information:	
Respiratory	Respiratory: Select...<br>If 'oxygen,' please select: ☐ PRN ☐ Continuous LPM:<br>Does patient require: ☐ CPAP ☐ BiPAP ☐ None<br>Settings:	
Infection control	Infection control: Select...<br>If 'other,' please specify:	Type of isolation:
**Skin Care**		**Issues to consider**
Skin	Does patient have pressure ulcers, surgical wounds, vascular, rash, or other skin issue: ☐ Yes ☐ No<br>If 'yes,' please describe:<br>Does patient require a wound vac? ☐ Yes ☐ No<br>Braden score:	Wound care:
**Other**		**Issues to consider**
PAC Stay	Did a provider in the hospital have a discussion regarding PAC? ☐ Yes ☐ No<br>If 'yes,' regarding what:<br>☐ Symptom management<br>☐ PAC resources<br>☐ Other:<br>If 'yes,' please describe:	

Equipment needed		
Labs and X-rays	Labs/X-rays needed:<br>Does patient have any labs/X-rays pending: ☐ Yes ☐ No<br>If 'yes, please describe how to obtain results:	
Vision and Hearing	Vision: ☐ Normal ☐ Impaired ☐ Blind<br>Hearing: ☐ Normal ☐ Impaired ☐ Deaf ☐ Hearing Aid: Selec<br>Personal items: ☐ Eyeglasses ☐ Dentures ☐ Other:	
Inpatient Consults		
Education provided		
Follow-up appointments		
Prepared By:	This report was prepared by: Select...<br>Prepared:	Pager number:
Highlights	Here are 1-3 issues that are most important regarding the care of this patient:	

Figure S2. Medication Management Form.

Reconciled Medication List for: Click here to enter text. **Last 4 of SSN:** Click here to enter text.

Allergies:

Completed by: _________________ **Contact number:** __________________

Updated: Click here to enter a date.

Pre-Hospital Medications	Action	Hospital Discharge Medications to Be Given at Post-Acute Care	Indication	Comments	Last dose

Blood glucose/Insulin

On day of hospital discharge:

	Breakfast	Lunch	Dinner	Bedtime
Blood glucose (mg/dL)				
Insulin scheduled (units)				
Insulin prn (units)				

On day prior to hospital discharge:

	Breakfast	Lunch	Dinner	Bedtime
Blood glucose (mg/dL)				
Insulin scheduled (units)				
Insulin prn (units)				

CHF Diuretics

Target (Dry) weight:

Instructions on daily weights:

	2 days prior to discharge	Day prior to discharge	Day of discharge
Creatinine			
Weight (lbs)			
Furosemide dose (mg)			

Anticoagulation:

- **Indication:** **Goal:** **Duration:**
- **Regimen:** Initiated on MM/DD/YY
    - Warfarin XXmg at bedtime
- **Recent INR:**
- **Other Labs:** Hgb XX, Hct XX, Platelet XX (MM/DD/YY)

Time invested:
